# Asymmetric localization of natural antisense RNA of neuropeptide sensorin in *Aplysia* sensory neurons during aging and activity

**DOI:** 10.3389/fgene.2014.00084

**Published:** 2014-04-22

**Authors:** Beena M. Kadakkuzha, Xin-An Liu, Maria Narvaez, Alexandra Kaye, Komolitdin Akhmedov, Sathyanarayanan V. Puthanveettil

**Affiliations:** Department of Neuroscience, The Scripps Research InstituteJupiter, FL, USA

**Keywords:** memory, nocoding RNA, antisense RNA, aging, *Aplysia*, neural circuitry

## Abstract

Despite the advances in our understanding of transcriptome, regulation and function of its non-coding components continue to be poorly understood. Here we searched for natural antisense transcript for sensorin (NAT-SRN), a neuropeptide expressed in the presynaptic sensory neurons of gill-withdrawal reflex of the marine snail *Aplysia californica*. Sensorin (SRN) has a key role in learning and long-term memory storage in *Aplysia*. We have now identified NAT-SRN in the central nervous system (CNS) and have confirmed its expression by northern blotting and fluorescent RNA *in situ* hybridization. Quantitative analysis of NAT-SRN in micro-dissected cell bodies and processes of sensory neurons suggest that NAT-SRN is present in the distal neuronal processes along with sense transcripts. Importantly, aging is associated with reduction in levels of NAT-SRN in sensory neuron processes. Furthermore, we find that forskolin, an activator of CREB signaling, differentially alters the distribution of SRN and NAT-SRN. These studies reveal novel insights into physiological regulation of natural antisense RNAs.

## Introduction

Recent high-throughput transcriptome studies have revealed widespread and extensive overlaps between genes and transcripts encoded on both strands of the genomic sequence. This overlapping gene organization, which produces sense-antisense transcript pairs, is capable of affecting regulatory cascades through established mechanisms. Natural antisense transcripts (NATs) are transcribed from the opposite strand to a protein coding or sense strand in the chromatin. Recent studies have provided ample evidence that more than 70% of the mammalian genome have antisense transcription potential.

Antisense transcription has been recognized for roles in gene regulation involving degradation of the corresponding sense transcripts (RNA interference), as well as gene silencing at the chromatin level (Faghihi and Wahlestedt, 2009; Pelechano and Steinmetz, 2013). Gene expression profiling studies show frequent concordant regulation of sense-antisense transcript pairs though there is clear evidence of discordant regulation, leading to significant physiological outcomes such as neurodegenerative diseases (Kadakkuzha et al., 2013). It has been shown that experimental modulation of an antisense transcript RNA can change the expression of sense transcript, supporting the role of antisense transcription to control of transcriptional outputs in higher animals (Katayama et al., 2005; Modarresi et al., 2011).

Despite efforts to unravel the specific role(s) of wide spread antisense transcription in recent years, the functional significance of NATs and their physiological regulation remains poorly understood. However, the significant presence of NATs in the central nervous system suggests their potential role in brain function. The role of NATs in establishing memory formation has been suggested in *Lymnaea* where axonal transport of NAT, described as antiNOS-2 RNA, is regulated by classical conditioning. AntiNOS-2 RNA negatively regulates the neurotransmitter nitric oxide (NO), a key transcript that plays an important role in the early stages of learning and memory formation (Korneev et al., 2013). It is not clear whether classical conditioning will lead to a net enhancement of antiNOS2 expression in the cell body and processes of CGC neuron or a selective increase in expression in the periphery.

To investigate physiological mechanisms that regulate expression and subcellular distribution of NATs, we have explored the expression of a NAT transcribed against the mRNA encoding the peptide neurotransmitter sensorin and its physiological regulation in the sensory neurons (SN) of the marine mollusk *Aplysia californica*. For more than 50 years *Aplysia* has provided fundamental insights into the basic organization of neuronal functions. *Aplysia's* nervous system has large neurons, many of them can be uniquely identified and are associated with specific behaviors. These neurons can be isolated and cultured *in vitro* and they form circuits, which can be investigated at the molecular and cellular detail.

The cell-specific neuropeptide, sensorin (SRN), is expressed exclusively in SNs and transported to distal neurites (Brunet et al., 1991). However, the distribution of sensorin transcripts in the SN cell bodies change when it is co-cultured with a motor neuron (Hu et al., 2002, 2003). It has been shown that formation and stabilization of sensory neuron (SN)-motor neuron (MN) synapses are regulated upon the release of sensorin peptide from SNs (Hu et al., 2004). *In vitro*, SNs uniquely make synapses with their *in vivo* target motor neurons but not with their non-target motor neurons, providing an excellent model system to specifically study the effects of specific mRNAs in synapse formation and stabilization (Kandel, 2001).

**Figure 1:**
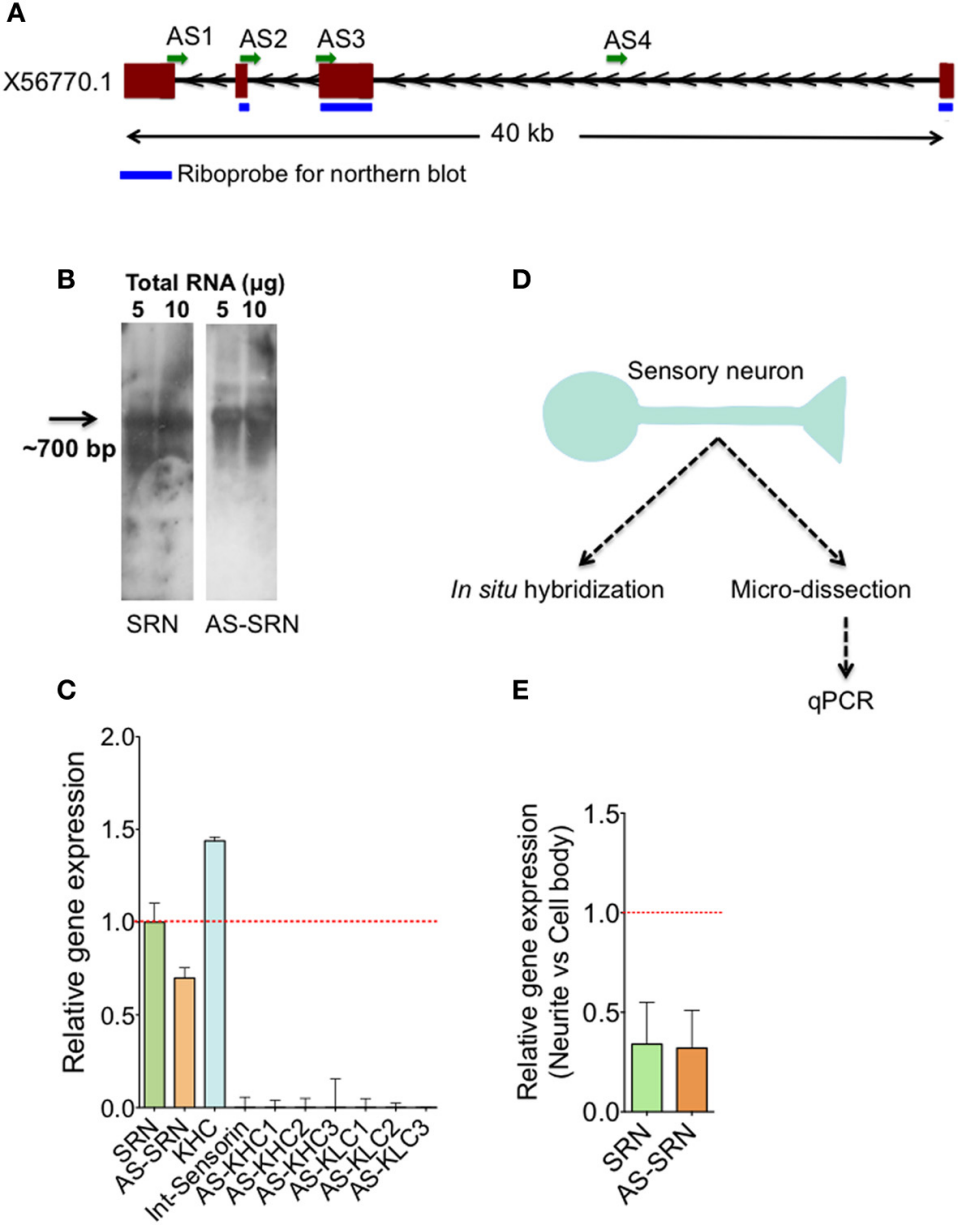
**Identification of natural antisense transcripts of neuropeptide sensorin**. Arrows indicate the direction of transcription. **(A)** The psc1 gene (EST X56770) coding for sensorin mRNA has four exons spanning ~40 KB on the scaffold 926 of *Aplysia*. AS1-4 represents the locations of qPCR amplicons that are used to detect AS transcript. **(B)** Northern blot detection of SRN and NAT-SRN from the total RNA of *Aplysia* CNS using strand specific ribo-probes. **(C)** qPCR analysis of SRN and NAT-SRN in RNA samples from *Aplysia* CNS. Primers that can amplify antisense direction of kinesin heavy chain, kinesin light chain and the primers to amplify intronic region of sensorin were used as negative controls. **(D)** Schematic diagram showing our strategy to study NAT-SRN in sensory neurons **(E)** qPCR analysis of SRN and NAT-SRN in the cell body and neurites of sensory neurons. Data was normalized to 18s rRNA levels. Error bars are SEM.

Figure 1D depicts the schematic diagram showing our strategy to study NAT-SRN in sensory neurons. Our analysis of gene expression using qPCR, northern blotting and single neuron quantitative PCR (qPCR) and fluorescent *in situ* hybridization (FISH) analyses have confirmed the expression of sense (SRN) and antisense RNAs (NAT-SRN) of neuropeptide sensorin in SNs of *Aplysia* gill withdrawal reflex. We then examined whether expression of NAT-SRN transcripts is regulated in SNs during aging and in response to forskolin, an activator of CREB (Seternes et al., 1999). We find that the expression levels and sub-cellular distribution of NAT-SRN are differentially altered during aging and neuronal activity.

## Materials and methods

### Ethics statement

The Institutional Biosafety Committee of The Scripps Research Institute (TSRI) has approved all of the experimental protocols (IBC Protocol 2010-019R1) described in this manuscript. Ethical approvals are not required for the research using invertebrate animals, such as the marine snail *Aplysia*.

### Animals, isolation of *Aplysia* neurons, and culture

*Aplysia californica* maintained under standard conditions (temperature, salinity, pH, food) at the National *Aplysia* Resource Facility (University of Miami Rosenstiel School of Medicine, Florida, USA) were used in the experiment. In our study, we used animals that correspond to two age groups (2 and 9 months old). Upon arrival in the laboratory, animals were kept in an aquarium at 16°C, under 12:12 light-dark conditions and was used for experiments within 2–3 days of arrival. Isolation of sensory neurons and culture were done as described earlier (Montarolo et al., 1986). Micro-dissection of cell body and processes were carried out as described earlier (Moccia et al., 2003; Moroz et al., 2006).

### Northern blot

A 415-bp DNA fragment corresponding to exons 1 and 2 of sensorin mRNA was cloned into TOPO Vector with dual promoters T7 and SP6 (Invitrogen, Cat. Number K4600-01) and then *in vitro* transcribed to prepare DIG labeled sense and antisense riboprobes using the DIG RNA Labeling Kit (SP6/T7) (Roche Diagnostics). The molecular sizes of riboprobes were confirmed by gel electrophoresis. Electrophoresis and transfer of the RNA was performed using DIG Northern Starter Kit (Roche Applied Science) and followed manufacturer's protocols. Briefly, 5 and 10 μg of total RNA from *Aplysia* was run on 1% agarose gel, transferred to a positive nitrocellulose membrane following hybridization with strand-specific riboprobes at 68°C overnight with ExpressHyb.

### RNA extraction and reverse transcription

Total RNA was extracted from the *Aplysia* CNS using the standard RNA Trizol extraction method and dissolved in nuclease-free water. For the preparation of RNA from SNs, cell body and neurites were separated by manual micro-dissection followed by RNA extraction using Trizol. RNAs were amplified once using MessageAmp™ II aRNA Amplification Kit following manufacturer's instructions. For the CNS samples 1 μg of the total RNA, and for the cell body and neurites, 500 ng of RNAs were used for reverse transcription using qScript cDNA synthesis mix.

### Quantitative real-time PCR

Primer pairs for SRN, NAT-SRN, and ApKHC1 and ApKLC2 were designed using the Primer3 program (http://bioinfo.ut.ee/primer3-0.4.0/) based on cDNA and genome sequences listed in UCSC genome browser database (http://genome.ucsc.edu/) (Figure 1A). While designing, primers steps are taken to avoid the amplification of multiple targets. Antisense primers were designed based on the exon boundaries of sense transcript to avoid potential amplification of the sense transcripts in the antisense detection samples. 2 μl of the cDNA synthesized was used for qPCR following the protocol described earlier (Kadakkuzha et al., 2013; Akhmedov et al., 2014). Briefly, 10 μl reactions contained 2 μl of cDNA, 8 μl of a qPCR master mix containing 2 μl of H_2_O, 5 μl of 2X SYBR Green master mix, and 1.0 μl of 10 μM (each) forward and reverse primer. The reaction was carried out in a 7900HT Fast Real-Time PCR System (Applied Biosystems Carlsbad, CA) under the following conditions: 95°C for 10 min, followed by 40 cycles of 95°C for 15 s, 60°C for 1 min. Five biological replicates and four technical replicates for each biological replicate were used in the qPCR. Quantification of the target transcripts was normalized to the *Aplysia*18S reference gene using the Pfaffl method (Pfaffl, 2001). Data are shown as mean ± s.e.m. Statistical analysis was performed using Prism (GraphPad Software). Student's *t*-test or ANOVA followed by Bonferroni's test were used as appropriate where ^*^P-value < 0.05, ^**^P-value < 0.01, ^***^P-value < 0.001.

### Preparation of dig labeled ribo probes

DIG labeled sense and antisense ribo probes for *in situ* hybridization probes were prepared by *in vitro* transcription of cDNA templates by using SP6 or T7 RNA polymerase. 415 nt long coding region of sensorin was prepared by PCR using *Aplysia* abdominal ganglion cDNA as a template and sensorin specific PCR primers and ligated to pCRII-TOPO Vector with dual promoters T7 and SP6 (Invitrogen, Cat. Number K4600-01). The Vector with the sensorin DNA was linearized with EcoR V (New England Biolab) for transcription using SP6 RNA polymerase to generate antisense probes and the corresponding sense probes were produced by linearizing with BamH I (New England Biolab) and transcribing with T7 RNA polymerase. A small aliquot (2 μ l) was run on 1.5% agarose gel to confirm the integrity of RNA probes.

### Fluorescent *in situ* hybridization (fish) and imaging analysis

DIG labeled RNA probes were prepared and *in situ* hybridization analysis of sensory neurons were carried out as described in (Puthanveettil et al., 2013). Images were acquired using a Zeiss LSM 780 confocal microscope system with 10X/63X objective; only projection images are shown. Mean fluorescence intensities were measured using NIH IMAGE J and normalized intensities were calculated using the following equation:

Normalized intensity = Mean FISH intensity (SRN or NAT-SRN) − Mean background signal.

Distributions of β-tubulin protein in both cell body and neuritis were measured to identify any non-specific changes in protein expression associated with aging or forskolin treatment.

## Results

### Detection of sensorin sense and antisense transcripts in *Aplysia* cns

Existence of sensorin antisense transcript (NAT-SRN) was previously suspected while performing *in situ* hybridization analysis of expression of sensorin in sensory neurons using fluorescently labeled sense (S) and antisense (AS) riboprobes (unpublished data). Sensorin mRNA (SRN) is transcribed from psc1 gene on the reverse strand of scaffold 926 of *Aplysia* genome (available through UCSC genome browser), spanning 40 KB long region and contains four exons (Figure 1A). In order to further characterize the sense (S) and the NAT at the psc1 locus, we searched for the presence of the NATs by northern blot, qPCR and *in situ* hybridization. Using DIG labeled ribo probes to detect SRN and NAT-SRN, we first analyzed total RNAs from the central nervous system (CNS) by northern hybridization analysis. Figure 1B show hybridization signals for SRN and NAT-SRN corresponding to ~700 nucleotides suggesting that NAT-SRN transcripts are expressed in the CNS.

Using specific primers (Supplementary Table 1) that detect SRN and NAT-SRN cDNA, we then confirmed the presence of SRN and NAT-SRN by qPCR. Primers designed to amplify the intronic regions of the SRN gene were used as negative controls and to make sure that the products were not generated from genomic DNA. From multiple primers that we used we selected AS2 (Figure 1A) for further qPCR detection of NAT-SRN. Primers to detect putative NAT of *Aplysia* kinesin heavy chain (KHC1) and KLC2 (Supplementary Table 1) were used as additional controls in qPCRs (Figure 1C). Data was normalized to 18S rRNA levels. qPCR results showed that SRN was expressed at ~30% higher levels when compared to NAT-SRN levels in CNS (*p* = 0.0235, ANOVA, Figure 1C).

### NAT-SRN is expressed in processes of sensory neurons

Previous studies have shown that release of sensorin from the SN is required for both synapse formation and long-term facilitation (LTF) of SN to MN connections. Localization of sensorin is modulated by the formation of synapses between SN and its target motor neurons (Lyles et al., 2006). We examined the distribution of both SRN and NAT-SRN isolated sensory neurons by qPCR. Analysis of RNAs isolated from micro dissected cell bodies and processes (Figure 1C) showed that both SRN and NAT-SRN transcripts are present in the neurites however, their levels in the cell bodies are much higher than that of neurites [~2-fold decrease in expression of SRN and NAT-SRN transcripts in the neurites compared to cell body, Figure 1E, Student's *t*-test; *p* = 0.043 (SRN), *p* = 0.027 (NAT-SRN)]. As a control we examined expression of *Aplysia* KHC1 transcripts in SN neurites. Specific primers for KHC1 were unable to detect expression of antisense or sense transcripts in SN neurites.

### Expression of NAT-SRN change during aging

**Figure 2:**
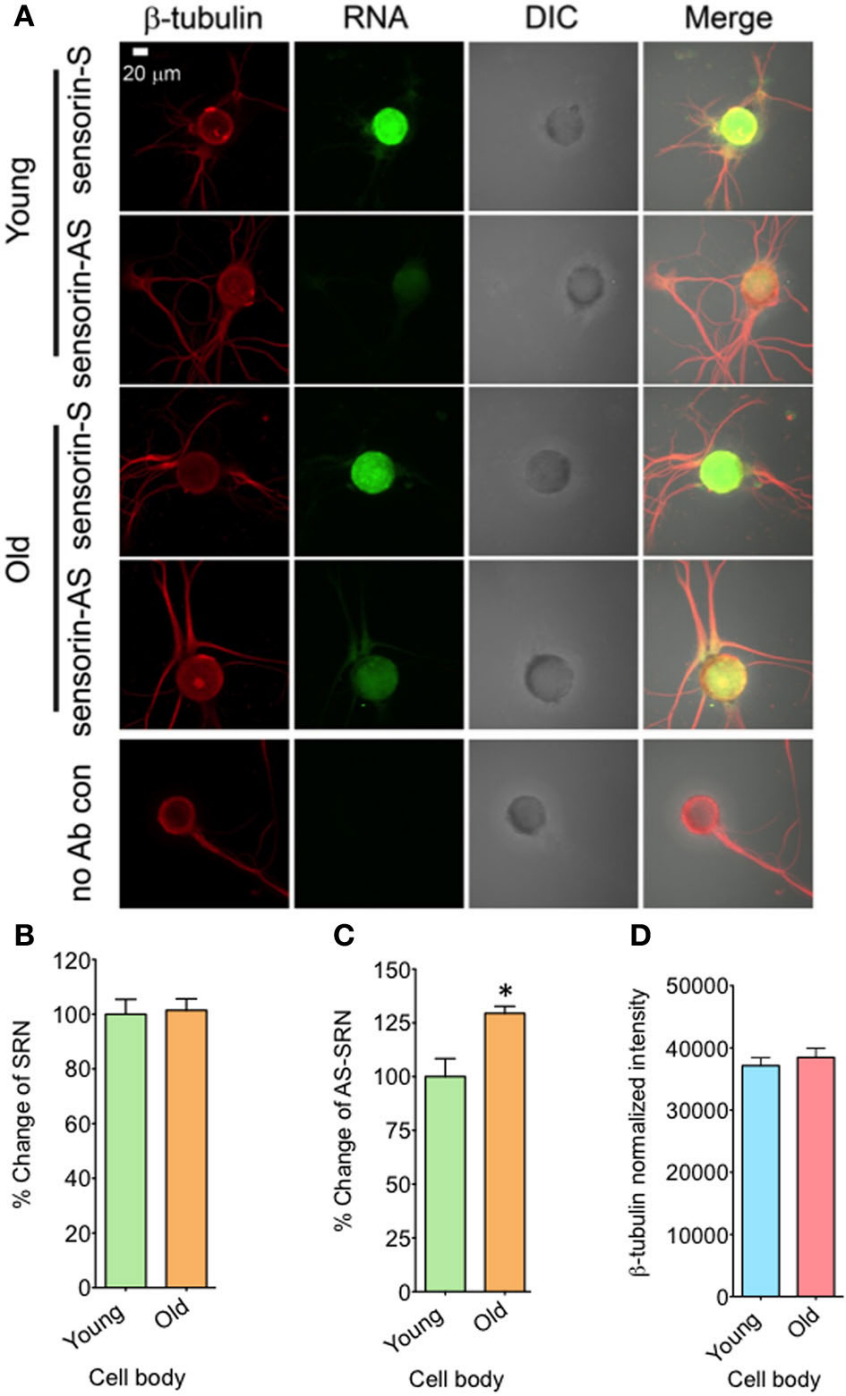
**Aging associated changes in expression of NAT-SRN in the cell body of *Aplysia* sensory neurons. (A)** Confocal projection images of sensorin RNA (Green) co-stained with β-tubulin protein (Red), DIC and merged images are shown. A no antibody (Ab) control was used as non-specific hybridization control. Scale bar, 20 μm; **(B,C)** are the percentage changes of SRN and NAT-SRN distribution in the cell body of DIV4-cultured sensory neurons from young (3 months old) and old (9 months old) groups of *Aplysia* using fluorescently labeled sense and antisense ribo probes, respectively. RNA *in situ* hybridization analysis of **(D)** is the normalized intensity of β-tubulin protein distribution in young and old neurites. Normalized mean fluorescence intensities measured using NIH ImageJ are shown in bar graphs. Error bars are SEM. Student's *t*-test was used to determine statistical significance. “^*^” is *p* < 0.05.

We next examined whether expression of NAT-SRN is physiologically regulated. We first studied whether aging is associated with a change in the distribution of NAT-SRN and looked at the distribution of SRN and NAT-SRN in sensory neurons cultured from 3 to 9 month old *Aplysia*. From FISH analysis it is evident that both SRN and NAT-SRN are present in the cell body of SNs from young and old animals (Figure 2A). The level of SRN transcript did not change notably in the cell bodies of neurons from young and old animals (Figure 2B, *N* = 4, Students' *t-test*, *p* = 0.8854) indicating that SRN transcript level in the cell body is not affected by aging. However, we observed a significant increase in the distribution of NAT-SRN in the cell body of SNs from old animals (Figure 2C, % increase: 25 ± 8, *N* = 4, Students' *t*-test, *p* = 0.0362). As an endogenous control we used the expression of β-tubulin and found that the β-tubulin protein levels in cell bodies of young and old neurons did not change significantly (*N* = 4, Students' *t*-test, *p* = 0.7504) (Figure 2D).

**Figure 3:**
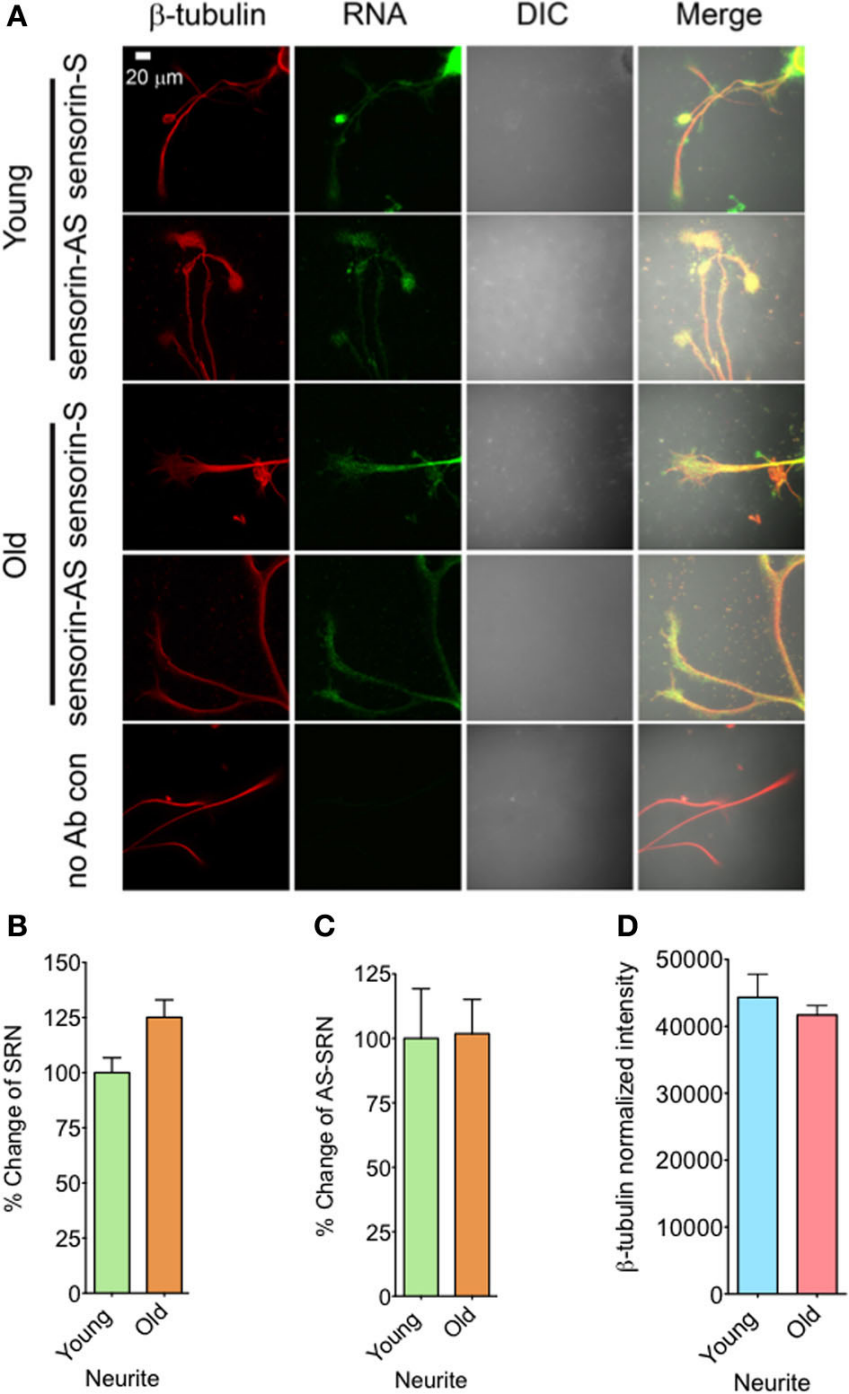
**Aging associated changes in expression of NAT-SRN in the neurites of *Aplysia* sensory neurons. (A)** Confocal projection images of sensorin RNA (Green) co-stained with β-tubulin protein (Red), DIC and merged. **(B,C)** are the percentage changes of SRN and NAT-SRN distribution in the neurites of DIV4-cultured sensory neurons, respectively. **(D)** is the normalized intensity of β-tubulin protein distribution in young and old neurites. Image analyses were performed as described in Figure 2.

We next analyzed the distribution of NAT-SRN in the processes of SNs cultured from young and old animals by FISH analysis and found moderate levels of SRN and NAT-SRN in the neurites of young and old animals (Figure 3A). Comparison of the mean intensities of FISH signals from young and old SRN and NAT-SRN after background signal subtraction suggested no change in the distribution of SRN transcript in the neurites of SNs from the young and old animals; an observation similar to what we found in the cell bodies described earlier (Figure 3B, *N* = 4, Students' *t*-test, *p* = 0.0864). Also, we did not observe any significant change in the level of NAT-SRN in the neurites of SNs from old animals when compared to young animals (Figure 3C, *N* = 4, Students' *t*-test, *p* = 0.9403). The endogenous control β-tubulin protein levels in processes of young and old neurons did not change significantly (*N* = 4, Students' *t*-test, *p* = 0.7701) (Figure 3D). These results suggest that the expression and localization of NAT-SRN transcripts are regulated during aging.

### Forskolin exposure enhance transcription of NAT-SRN in both cell bodies and neurites

**Figure 4:**
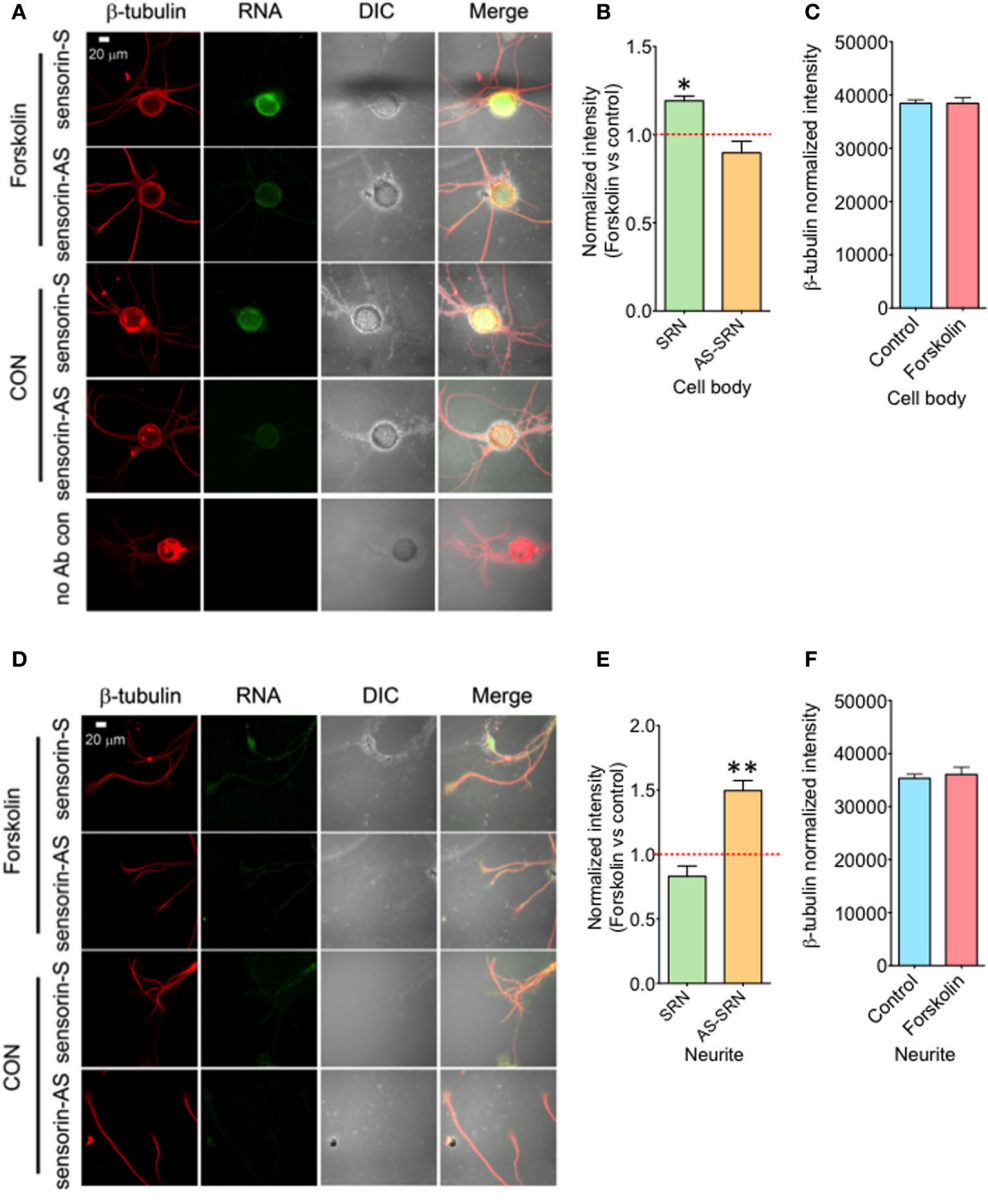
**Forskolin exposure enhances expression of NAT-SRN in *Aplysia* sensory neurons**. Sensory neurons of mature *Aplysia* were cultured *in vitro* and were treated with forskolin (50 μ M, 30 min) on DIV 4. **(A)** Confocal projection images of FISH analysis of sensorin RNA (SRN) (Green) and **(D)** that of AS-sensorin RNA (NAT-SRN) (Green) co-stained with β-tubulin protein (Red), as well as DIC images are shown. A no antibody control was used as non-specific hybridization control. Scale bar, 20 μm. **(B,E)** show the change in expression levels of SRN and NAT-SRN in forskolin treated neurons when compared with vehicle treated control neurons in cell body and neuritis, respectively. **(C,F)** are the normalized intensity of β-tubulin protein levels in control and forskolin treated cell body and neuritis, respectively. Normalized mean fluorescence intensities measured using NIH ImageJ are shown in bar graphs. Error bars are SEM. Student's *t*-test was used to determine statistical significance. “^*^” is *p* < 0.05, “^**^” is *p* < 0.01.

We next studied whether NAT-SRN levels could be regulated by forskolin, an activator of cAMP-CREB signaling. SN cultures (4 DIV) were treated with 50 μM forskolin for 30 min (Puthanveettil et al., 2008) and fixed for FISH analysis (Figure 4A). Vehicle treated SNs were used as controls. Analysis of mean fluorescence intensities of expression of SRN and NAT-SRN suggest that forskolin treatment induced 25% increase in the expression level of SRN in the cell body (Figure 4B) but no change in NAT-SRN level (Student's *t*-test; *p* = 0.0007). Interestingly, there was no change in the level of SRN in the neurites of SNs treated with forskolin within 30 min of treatment but the NAT-SRN level was increased by 50% after forskolin treatment in the neurites (Figures 4D,E; Student's *t*-test; *p* = 0.001). β-tubulin protein levels in the cell body (Figure 4C) and processes (Figure 4F) of control and forskolin treated neurons did not change significantly (*N* = 4, Students' *t*-test, *p* = 0.7225 and 0.6712, respectively).

## Discussion

Advances in sequencing methodologies have led to sequencing of several genomes and transcriptomes shedding light on the non-coding component of the genome. These large-scale sequencing studies have resulted in cataloging of thousands of non-coding RNAs in a variety of organisms (Katayama et al., 2005; Li et al., 2013). We have now begun to understand the functional relevance of transcription of non-coding RNAs and physiological mechanisms that regulate transcription of non-coding RNAs. Non-coding RNAs include miRNAs, piRNAs, tRNAs, rRNAs, snRNAs, large non-coding RNAs, and natural antisense RNAs. NAT are intriguing because these RNAs have transcripts complementary to other RNA transcripts. Several regulatory roles for NATs have been suggested including RNA interference, genomic imprinting, and alternate splicing (Zhang et al., 2007; Faghihi and Wahlestedt, 2009; Werner, 2013; Wight and Werner, 2013).

Recent studies have demonstrated that NATs could downregulate expression of its complementary transcripts. For example, in the rice plant, NATs regulate expression of a protein important for phosphate homeostasis (Jabnoune et al., 2013). Similarly NAT of interleukin (IL) 1 beta) (Lu et al., 2013), iNOS (inducible nitric oxide synthase) (Yoshigai et al., 2013), and ubiquitin c-terminal hydrolase (uch) (Carrieri et al., 2012), Huntington's disease (Chung et al., 2011) suppress expression of their complementary transcripts. Importantly, inhibition of expression of NATs has resulted in upregulation of gene specific transcription (Modarresi et al., 2012). Recently, (Velmeshev et al., 2013) showed that 40% of loci previously implicated in autism spectrum disorders express NATs. These NATs are expressed in specific brain regions.

We are only beginning to understand the physiological processes and mechanisms that regulate expression of NATs. In plants, drought alters expression of NATs (Lembke et al., 2012). Similarly during beta amyloid induced apoptosis, NAT of Rad18 gene become upregulated (Parenti et al., 2007). Also, during corticogenesis in mouse brain the expression of NAT of Nrgn and CamK2n1 are regulated (Ling et al., 2011). Additionally, it has been shown that sense-antisense transcript pairs are present in synaptoneurosomes (Smalheiser et al., 2008).

Despite these elegant studies, we still do not know whether and how NATs are regulated in specific neural circuitries. To address this we used well-characterized neural circuitry in *Aplysia*, the sensory-motor neurons of gill withdrawal reflex (Kandel, 2001) and studied potential NAT of neuropeptide sensorin (NAT-SRN) in sensory neurons. Neuropeptide sensorin is expressed in presynaptic sensory neurons and is important for LTF of sensory to motor neuron synapses (Brunet et al., 1991; Schacher et al., 1999). Sensorin RNA is transported to synapses (Schacher et al., 1999; Moccia et al., 2003) and translated in response to repeated 5-HT stimulation (Wang et al., 2009). To search for putative NAT-SRN, we first analyzed *Aplysia* genome and designed primers to prepare sense and antisense probes for northern analysis. We find that NAT-SRN is expressed in *Aplysia* CNS and that it probably has a similar molecular weight as compared to complementary sense transcript. We next confirmed the expression of NAT-SRN by qPCR analysis of CNS and micro-dissected cell body and processes of individual SNs. Having confirmed the expression of NAT-SRN in SNs, we then asked two questions: (a) whether NAT-SRN is physiologically regulated and (b) whether NAT-SRN expression could be regulated by cellular activities that elicit specific signaling pathways leading to memory storage.

To understand physiological regulation of NAT-SRN, we first determined whether its expression changes during aging and whether aging causes changes in subcellular distribution of NAT-SRN. Importantly, aging associated changes in specific NATs are poorly understood. We studied two age groups, young and old *Aplysia*. Our FISH analyses suggest that the amount of NAT-SRN increases in cell body of old neurons when compared to corresponding sense transcripts. However, the NAT-SRN level is decreased in neurites of sensory neurons cultured from old animals.

To determine whether specific cellular activities might regulate expression of NAT-SRN, we measured changes in expression of NAT-SRN in response to forskolin, an activator of cAMP-PKA-CREB pathway important for long-term memory storage (Kandel, 2001). Recently (Korneev et al., 2013) have shown that classical conditioning of *Lymnaea* changes in the expression of NAT of nitric oxide synthase. Consistent with the idea that NATs could be physiologically regulated, we find that immediately after forskolin treatment, there is an increase in SRN transcripts in the cell body. However, forskolin did not cause an increase in the expression of NAT-SRN in the cell body. Interestingly we find that forskolin exposure resulted in a significant increase in NAT-SRN transcripts in the neurites.

The differential subcellular localization of sense and NAT-SRN during aging and in response to forskolin treatment suggest that there might be specific mechanisms that mediate differential expression and transport of SRN and NAT-SRN transcripts. We consider three possible mechanisms: (a) regulation of transcription of NAT-SRN or (b) degradation of NAT-SRN in specific compartments, or (c) changes in axonal transport of NAT-SRN to neurites. It has been shown that expression of NATs could be regulated by epigenetic mechanisms (Conley and Jordan, 2012). We find that exposure to forskolin cause a rapid increase in sense transcripts in cell body and increase in NAT-SRN in neurites. Our observation that forskolin did not cause upregulation of NAT-SRN in the cell body suggests the possibility that transcription of NAT-SRN is not regulated by CREB. We have previously shown that (Puthanveettil et al., 2008) forskolin treatment cause a rapid increase in kinesin mRNA levels, the molecular motor that mediates axonal transport, in sensory neurons. However, it is yet to be determined whether NAT-SRN is transported by kinesin and whether increase in NAT-ARN in SN neurites correlate with enhanced expression of kinesin mRNAs.

In summary, we have identified expression of natural antisense RNA of sensorin, an important neuropeptide involved in memory storage. We find that its subcellular localization in sensory neurons is differentially regulated during aging and in response to activation of cAMP-PKA-CREB pathway. We now provide evidence that NATs could be differentially regulated in different sub-cellular compartments. Further studies are required to delineate mechanisms and understand physiological implications of such regulation.

### Conflict of interest statement

The authors declare that the research was conducted in the absence of any commercial or financial relationships that could be construed as a potential conflict of interest.

## References

[B1] Akhmedov K. Kadakkuzha B. M. Puthanveettil S. V. (2014). Aplysia Ganglia preparation for electrophysiological and molecular analyses of single neurons. J. Vis. Exp. e51075 10.3791/51075 24457225 PMC4089395

[B2] Brunet J. F. Shapiro E. Foster S. A. Kandel E. R. Iino Y. (1991). Identification of a peptide specific for aplysia sensory neurons by Pcr-Based differential screening. Science 252, 856–859 10.1126/science.1840700 1840700

[B3] Carrieri C. Cimatti L. Biagioli M. Beugnet A. Zucchelli S. Fedele S. (2012). Long non-coding antisense RNA controls Uchl1 translation through an embedded SINEB2 repeat. Nature 491, 454–457 10.1038/nature11508 23064229

[B4] Chung D. W. Rudnicki D. D. Yu L. Margolis R. L. (2011). A natural antisense transcript at the Huntington's disease repeat locus regulates HTT expression. Hum. Mol. Genet. 20, 3467–3477 10.1093/hmg/ddr263 21672921 PMC3153309

[B5] Conley A. B. Jordan I. K. (2012). Epigenetic regulation of human cis-natural antisense transcripts. Nucleic Acids Res. 40, 1438–1445 10.1093/nar/gkr1010 22371288 PMC3287164

[B6] Faghihi M. A. Wahlestedt C. (2009). Regulatory roles of natural antisense transcripts. Nat. Rev. Mol. Cell Biol. 10, 637–643 10.1038/nrm2738 19638999 PMC2850559

[B7] Hu J. Y. Goldman J. Wu F. Schacher S. (2004). Target-dependent release of a presynaptic neuropeptide regulates the formation and maturation of specific synapses in Aplysia. J. Neurosci. 24, 9933–9943 10.1523/JNEUROSCI.3329-04.2004 15525778 PMC6730238

[B8] Hu J. Y. Meng X. Schacher S. (2002). Target interaction regulates distribution and stability of specific mRNAs. J. Neurosci. 22, 2669–2678 11923432 10.1523/JNEUROSCI.22-07-02669.2002 PMC6758340

[B9] Hu J. Y. Meng X. Schacher S. (2003). Redistribution of syntaxin mRNA in neuronal cell bodies regulates protein expression and transport during synapse formation and long-term synaptic plasticity. J. Neurosci. 23, 1804–1815 12629184 10.1523/JNEUROSCI.23-05-01804.2003 PMC6741965

[B10] Jabnoune M. Secco D. Lecampion C. Robaglia C. Shu Q. Poirier Y. (2013). A Rice cis-natural antisense RNA acts as a translational enhancer for its cognate mRNA and contributes to phosphate homeostasis and plant fitness. Plant Cell 25, 4166–4182 10.1105/tpc.113.116251 24096344 PMC3877805

[B11] Kadakkuzha B. M. Akhmedov K. Capo T. R. Carvalloza A. C. Fallahi M. Puthanveettil S. V. (2013). Age-associated bidirectional modulation of gene expression in single identified R15 neuron of Aplysia. BMC Genomics 14:880 10.1186/1471-2164-14-880 24330282 PMC3909179

[B12] Kandel E. R. (2001). The molecular biology of memory storage: a dialogue between genes and synapses. Science 294, 1030–1038 10.1126/science.1067020 11691980

[B13] Katayama S. Tomaru Y. Kasukawa T. Waki K. Nakanishi M. Nakamura M. (2005). Antisense transcription in the mammalian transcriptome. Science 309, 1564–1566 10.1126/science.1112009 16141073

[B14] Korneev S. A. Kemenes I. Bettini N. L. Kemenes G. Staras K. Benjamin P. R. (2013). Axonal trafficking of an antisense RNA transcribed from a pseudogene is regulated by classical conditioning. Sci. Rep. 3, 1027 10.1038/srep01027 23293742 PMC3537157

[B15] Lembke C. G. Nishiyama M. Y. Jr. Sato P. M. De Andrade R. F. Souza G. M. (2012). Identification of sense and antisense transcripts regulated by drought in sugarcane. Plant Mol. Biol. 79, 461–477 10.1007/s11103-012-9922-1 22610347 PMC3369129

[B16] Li J. Xuan Z. Liu C. (2013). Long non-coding RNAs and complex human diseases. Int. J. Mol. Sci. 14, 18790–18808 10.3390/ijms140918790 24036441 PMC3794807

[B17] Ling K. H. Hewitt C. A. Beissbarth T. Hyde L. Cheah P. S. Smyth G. K. (2011). Spatiotemporal regulation of multiple overlapping sense and novel natural antisense transcripts at the Nrgn and Camk2n1 gene loci during mouse cerebral corticogenesis. Cereb. Cortex 21, 683–697 10.1093/cercor/bhq141 20693275

[B18] Lu J. Wu X. Hong M. Tobias P. Han J. (2013). A potential suppressive effect of natural antisense IL-1beta RNA on lipopolysaccharide-induced IL-1beta expression. J. Immunol. 190, 6570–6578 10.4049/jimmunol.1102487 23677478 PMC3690360

[B19] Lyles V. Zhao Y. Martin K. C. (2006). Synapse formation and mRNA localization in cultured Aplysia neurons. Neuron 49, 349–356 10.1016/j.neuron.2005.12.029 16446139

[B20] Moccia R. Chen D. Lyles V. Kapuya E., E, Y. Kalachikov S. (2003). An unbiased cDNA library prepared from isolated Aplysia sensory neuron processes is enriched for cytoskeletal and translational mRNAs. J. Neurosci. 23, 9409–9417 14561869 10.1523/JNEUROSCI.23-28-09409.2003 PMC6740582

[B21] Modarresi F. Faghihi M. A. Lopez-Toledano M. A. Fatemi R. P. Magistri M. Brothers S. P. (2012). Inhibition of natural antisense transcripts *in vivo* results in gene-specific transcriptional upregulation. Nat. Biotechnol. 30, 453–459 10.1038/nbt.2158 22446693 PMC4144683

[B22] Modarresi F. Faghihi M. A. Patel N. S. Sahagan B. G. Wahlestedt C. Lopez-Toledano M. A. (2011). Knockdown of BACE1-AS nonprotein-coding transcript modulates beta-amyloid-related hippocampal neurogenesis. Int. J. Alzheimers Dis. 2011, 929042 10.4061/2011/929042 21785702 PMC3139208

[B23] Montarolo P. G. Goelet P. Castellucci V. F. Morgan J. Kandel E. R. Schacher S. (1986). A critical period for macromolecular synthesis in long-term heterosynaptic facilitation in Aplysia. Science 234, 1249–1254 10.1126/science.3775383 3775383

[B24] Moroz L. L. Edwards J. R. Puthanveettil S. V. Kohn A. B. Ha T. Heyland A. (2006). Neuronal transcriptome of Aplysia: neuronal compartments and circuitry. Cell 127, 1453–1467 10.1016/j.cell.2006.09.052 17190607 PMC4024467

[B25] Parenti R. Paratore S. Torrisi A. Cavallaro S. (2007). A natural antisense transcript against Rad18, specifically expressed in neurons and upregulated during beta-amyloid-induced apoptosis. Eur. J. Neurosci. 26, 2444–2457 10.1111/j.1460-9568.2007.05864.x 17970741

[B26] Pelechano V. Steinmetz L. M. (2013). Gene regulation by antisense transcription. Nat. Rev. Genet. 14, 880–893 10.1038/nrg3594 24217315

[B27] Pfaffl M. W. (2001). A new mathematical model for relative quantification in real-time RT-PCR. Nucleic Acids Res. 29, e45 10.1093/nar/29.9.e45 11328886 PMC55695

[B28] Puthanveettil S. V. Antonov I. Kalachikov S. Rajasethupathy P. Choi Y. B. Kohn A. B. (2013). A strategy to capture and characterize the synaptic transcriptome. Proc. Natl. Acad. Sci. U.S.A. 110, 7464–7469 10.1073/pnas.1304422110 23589870 PMC3645558

[B29] Puthanveettil S. V. Monje F. J. Miniaci M. C. Choi Y. B. Karl K. A. Khandros E. (2008). A new component in synaptic plasticity: upregulation of kinesin in the neurons of the gill-withdrawal reflex. Cell 135, 960–973 10.1016/j.cell.2008.11.003 19041756 PMC2635114

[B30] Schacher S. Wu F. Panyko J. D. Sun Z. Y. Wang D. (1999). Expression and branch-specific export of mRNA are regulated by synapse formation and interaction with specific postsynaptic targets. J. Neurosci. 19, 6338–6347 10414963 10.1523/JNEUROSCI.19-15-06338.1999 PMC6782793

[B31] Seternes O. M. Johansen B. Moens U. (1999). A dominant role for the Raf-MEK pathway in forskolin, 12-O-tetradecanoyl-phorbol acetate, and platelet-derived growth factor-induced CREB (cAMP-responsive element-binding protein) activation, uncoupled from serine 133 phosphorylation in NIH 3T3 cells. Mol. Endocrinol. 13, 1071–1083 10.1210/mend.13.7.0293 10406459

[B32] Smalheiser N. R. Lugli G. Torvik V. I. Mise N. Ikeda R. Abe K. (2008). Natural antisense transcripts are co-expressed with sense mRNAs in synaptoneurosomes of adult mouse forebrain. Neurosci. Res. 62, 236–239 10.1016/j.neures.2008.08.010 18812194 PMC2597790

[B33] Velmeshev D. Magistri M. Faghihi M. A. (2013). Expression of non-protein-coding antisense RNAs in genomic regions related to autism spectrum disorders. Mol. Autism. 4, 32 10.1186/2040-2392-4-32 24007600 PMC3851999

[B34] Wang D. O. Kim S. M. Zhao Y. Hwang H. Miura S. K. Sossin W. S. (2009). Synapse- and stimulus-specific local translation during long-term neuronal plasticity. Science 324, 1536–1540 10.1126/science.1173205 19443737 PMC2821090

[B35] Werner A. (2013). Biological functions of natural antisense transcripts. BMC Biol. 11:31 10.1186/1741-7007-11-31 23577602 PMC3626547

[B36] Wight M. Werner A. (2013). The functions of natural antisense transcripts. Essays Biochem. 54, 91–101 10.1042/bse0540091 23829529 PMC4284957

[B37] Yoshigai E. Machida T. Okuyama T. Mori M. Murase H. Yamanishi R. (2013). Citrus nobiletin suppresses inducible nitric oxide synthase gene expression in interleukin-1beta-treated hepatocytes. Biochem. Biophys. Res. Commun. 439, 54–59 10.1016/j.bbrc.2013.08.029 23958298

[B38] Zhang Y. Li J. Kong L. Gao G. Liu Q. R. Wei L. (2007). NATsDB: natural antisense transcripts database. Nucleic Acids Res. 35, D156–D161 10.1093/nar/gkl782 17082204 PMC1635336

